# Site-Specifically Labeled Immunoconjugates for Molecular Imaging—Part 1: Cysteine Residues and Glycans

**DOI:** 10.1007/s11307-015-0919-4

**Published:** 2016-01-11

**Authors:** Pierre Adumeau, Sai Kiran Sharma, Colleen Brent, Brian M. Zeglis

**Affiliations:** Department of Chemistry and Biochemistry, Hunter College and the Graduate Center of the City University of New York, 413 East 69th Street, New York, NY 10021 USA; Department of Radiology, Memorial Sloan Kettering Cancer Center, 1275 York Avenue, New York, NY10065 NY USA

**Keywords:** Positron emission tomography, PET, Single photon emission computed tomography, SPECT, Fluorescence imaging, Near-infrared fluorescence imaging, Optical imaging, Click chemistry, Site-specific conjugation, Site-selective conjugation, Bioconjugation, Bioorthogonal chemistry, Glycoengineering, Protein engineering, Antibody, Antibody fragment, Immunoglobulins, Cysteine, Maleimide, Glycans

## Abstract

Due to their remarkable selectivity and specificity for cancer biomarkers, immunoconjugates have emerged as extremely promising vectors for the delivery of diagnostic radioisotopes and fluorophores to malignant tissues. Paradoxically, however, these tools for precision medicine are synthesized in a remarkably imprecise way. Indeed, the vast majority of immunoconjugates are created *via* the random conjugation of bifunctional probes (*e.g.*, DOTA-NCS) to amino acids within the antibody (*e.g.*, lysines). Yet antibodies have multiple copies of these residues throughout their macromolecular structure, making control over the location of the conjugation reaction impossible. This lack of site specificity can lead to the formation of poorly defined, heterogeneous immunoconjugates with suboptimal *in vivo* behavior. Over the past decade, interest in the synthesis and development of site-specifically labeled immunoconjugates—both antibody-drug conjugates as well as constructs for *in vivo* imaging—has increased dramatically, and a number of reports have suggested that these better defined, more homogeneous constructs exhibit improved performance *in vivo* compared to their randomly modified cousins. In this two-part review, we seek to provide an overview of the various methods that have been developed to create site-specifically modified immunoconjugates for positron emission tomography, single photon emission computed tomography, and fluorescence imaging. We will begin with an introduction to the structure of antibodies and antibody fragments. This is followed by the core of the work: sections detailing the four different approaches to site-specific modification strategies based on cysteine residues, glycans, peptide tags, and unnatural amino acids. These discussions will be divided into two installments: cysteine residues and glycans will be detailed in Part 1 of the review, while peptide tags and unnatural amino acids will be addressed in Part 2. Ultimately, we sincerely hope that this review fosters interest and enthusiasm for site-specific immunoconjugates within the nuclear medicine and molecular imaging communities.

## Introduction

**Fig. 1:**
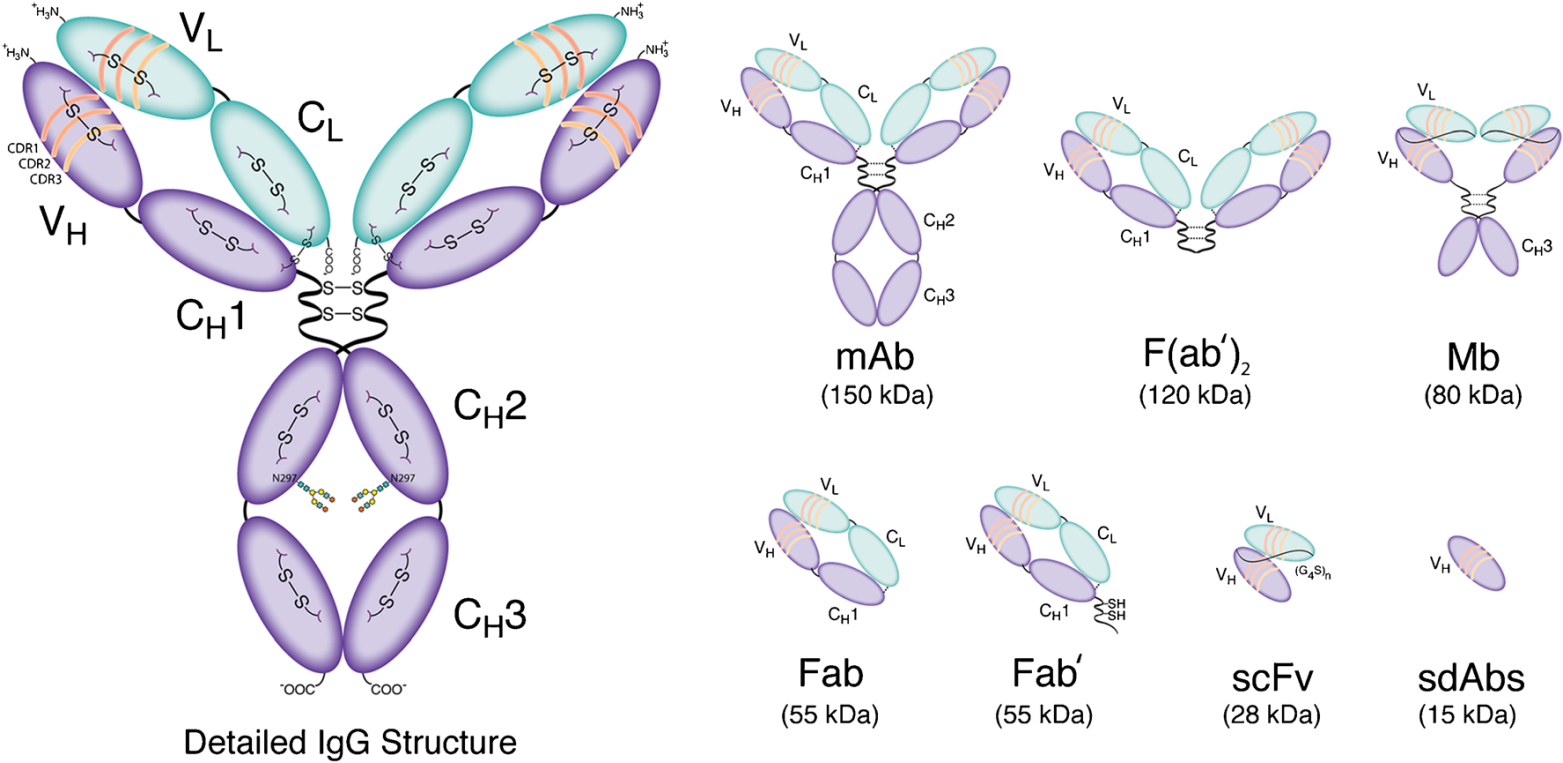
Detailed structural schematic of a full-length IgG as well as an assortment of antibody fragments.

Over the last three decades, medical imaging has revolutionized cancer care, providing clinicians with the means to noninvasively acquire anatomical, functional, and biological information about tumors. Due to their remarkable affinity and specificity for cancer biomarkers, antibodies—as well as an ever-growing array of antibody fragments—have played an increasingly important role in this field (Fig. 1) [1, 2]. Indeed, antibody conjugates bearing a wide range of reporters—ranging from Zr-89 for positron emission tomography (PET) to near-infrared fluorophores for optical imaging (OI)—have been successfully developed and translated to the clinic [3, 4].

**Fig. 2:**
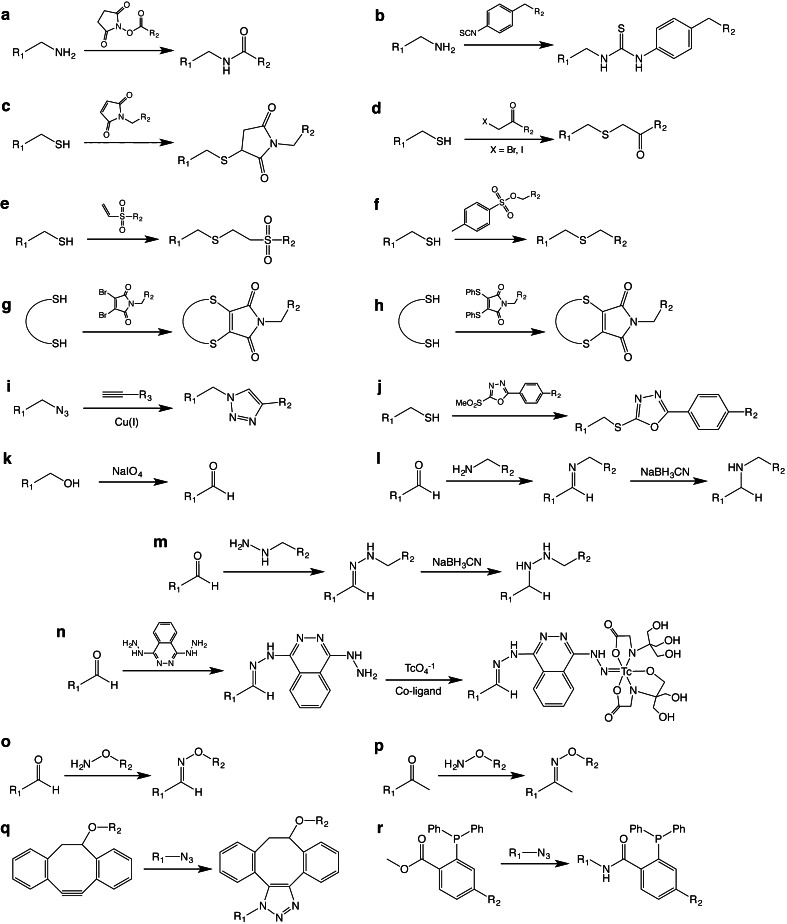
The basic chemical reactions underpinning the bioconjugation strategies discussed in this work.

**Fig. 3:**
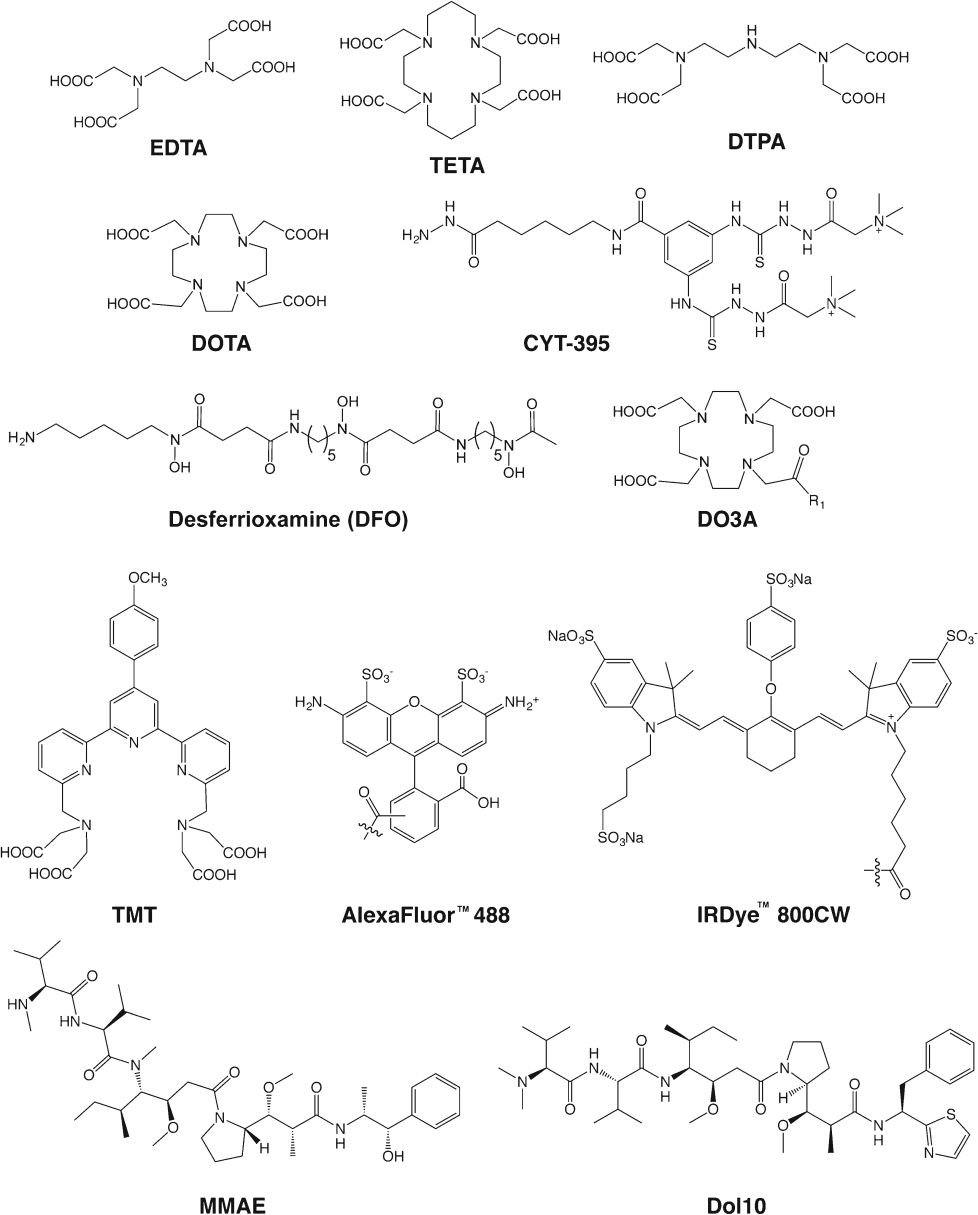
Selected chelators and cargoes used in the site-specifically labeled immunoconjugates discussed in this work.

Yet paradoxically, these agents designed to enable “precision medicine” are synthesized in a rather imprecise manner. At present, the vast majority of bioconjugation techniques rely on reactions between bifunctional probes and amino acids, typically lysines (Fig. 2a, b) [5–7]. For example, in the case of Zr-89-labeled antibodies for PET imaging, an isothiocyanate-bearing derivative of the Zr-89 chelator desferrioxamine (DFO-NCS; Fig. 3) is conjugated randomly to lysines in the immunoglobulin [6]. However, antibodies possess varying numbers of these residues distributed throughout their macromolecular structure. Thus, controlling the molecular location of these conjugation reactions and the number of conjugations per antibody is impossible.

These random bioconjugation approaches produce immunoconjugates that are poorly defined and heterogeneous on three different levels [8–10]. First, a single conjugation reaction using these methods will produce a product with a range of degrees of labeling. For example, the total population of an immunoconjugate with an average loading of 3 chelators/monoclonal antibody (mAb) will include subpopulations with degrees of labeling ranging from 0 to well above 3. Second, even immunoconjugates that possess identical degrees of labeling are likely to be regioisomers. If, for example, we assume an antibody has 40 available lysines, an immunoconjugate with a degree of labeling of 2 chelators/mAb is actually a mixture of up to 780 different regioisomers, while an immunoconjugate with a degree of labeling of 3 chelators/mAb is actually a mixture of over 10,000 different regioisomers! And third, random conjugation strategies present batch-to-batch reproducibility issues. Even if two batches of an immunoconjugate possess the same degree of labeling, it is extremely unlikely that these two batches are composed of the exact same mixture of regioisomers.

This heterogeneity should not be dismissed as an academic issue. Each regioisomer has a unique set of chemical, biological, and pharmacological traits. An antibody with a single fluorophore attached to a lysine in the C_H_3 region, for example, will likely exhibit *in vivo* pharmacokinetics different from that of an antibody bearing five fluorophores attached to lysines in the V_H_ and C_H_1 domains. Furthermore, without the ability to control the precise location of the conjugation reactions, cargoes may become appended to the antigen-binding domains of the antibody, thus impairing the immunoreactivity of the conjugate [11]. Taken together, these issues can have adverse effects on the *in vivo* performance of immunoconjugates, resulting in suboptimal pharmacokinetics, decreased accumulation in target tissues, and increased uptake in healthy tissues. There are logistical drawbacks to random bioconjugation methods as well. In the absent of precise control over the modification process, every new immunoconjugate must undergo extensive optimization, a process that can be costly, time-consuming, and tedious.

In response to these problems, the last decade has played witness to a great deal of research into the development of methodologies for the site-specific modification of antibodies [8, 12–16]. On the most basic level, the key to any site-specific bioconjugation strategy is *selectivity*. A variant of the cargo molecule—whether a chelator, fluorophore, drug, or prosthetic group—must be designed to react chemoselectively with a specific site or sites in the structure of the antibody. Effective site-specific bioconjugation strategies have been developed using a wide range of pathways to achieve chemoselectivity, including bioorthogonal organic transformations, click chemistry, and enzymatic reactions [17–22]. Regardless of the specifics, however, the end result in every case is straightforward: the creation of better defined, more homogeneous immunoconjugates.

Practically speaking, these site-specific modification strategies offer a number of important advantages over traditional random modification methods. First, site-specific approaches reproducibly yield better defined and more homogeneous immunoconjugates, simultaneously eliminating the problems of heterogeneity and irreproducibility created by random approaches. Second, because site-specific procedures enable the precise control over the molecular location of the conjugation reaction, these methods prevent the inadvertent attachment of cargoes to the antigen-binding domains of the antibody. Third, it is almost certain that regulatory agencies would look more favorably on well-defined, homogeneous immunoconjugates compared to the complex, heterogeneous mixtures of constructs created using random conjugation strategies. Finally, and perhaps most importantly, a number of intriguing reports have found that site-specifically modified immunoconjugates exhibit superior *in vivo* behavior to their traditionally synthesized cousins, boasting more favorable pharmacokinetics, higher uptake in target tissues, and lower background accumulation in healthy tissues [14, 23–27].

In this two-part review, it is our goal to provide an overview of the various methods that have been developed to create site-specifically modified immunoconjugates for PET, single photon emission computed tomography (SPECT), and fluorescence imaging. Furthermore, due to the advent of antibody fragments as smaller, more pharmacokinetically rapid alternatives to full-length IgGs, we have decided to include immunoconjugates based on these constructs as well [28, 29]. Given the tremendous amount of work to cover, we have divided this review into two parts. In Part 1, we will begin with an introduction to the structure of antibodies and antibody fragments, followed by detailed discussions of the site-specific modification strategies based on cysteine residues and glycans. In Part 2, we will shift our focus to site-specific bioconjugation approaches based on peptide tags and unnatural and noncanonical amino acids. In Part 2, we will also offer a broad overview of the advantages and disadvantages of the various approaches to conjugation as well as some rumination on the direction of the field as a whole. Importantly, there are a number of cases in which a given site-specific modification strategy *has* been used in the creation of an antibody-drug conjugates (ADCs) but *has not yet* been employed to create an immunoconjugate for imaging. In these cases, we have chosen to discuss the approach in question—if only briefly—in order to increase the breadth of this work and encourage the application of these methods to imaging agents. For readers specifically interested in the construction of ADCs, we recommend a few recent and extremely well-written reviews [8, 14, 16]. In addition, we have found a small number of reports detailing the creation of site-specifically labeled antibodies for *radioimmunotherapy*; given that the development of agents for nuclear imaging and targeted radiotherapy often go hand in hand, we have included these examples as well. Finally, we would also like to issue a small caveat. The development of site-specific antibody modification strategies is a rapidly growing field. We have tried to cover as many of the different approaches as we could find in the literature. However, it is all but certain that we have missed at least one report, most likely more. To the authors of these works, we humbly apologize in advance.

## Immunoglobulin Structure

### Antibodies

Discovered in the late nineteenth century as toxin-neutralizing agents in the blood of animals infected with diphtheria, antibodies are globular proteins produced by the immune system, hence the term “immunoglobulin” [30]. As seen in electron micrographs, antibodies are Y-shaped molecules with a bifurcated end joined to a stalk by a flexible hinge region (Fig. 1) [31]. The forked end consists of the antigen-binding fragments—*i.e.*, the Fab region—that define the specificity of the antibody for its antigen target, while the stalk—*i.e.*, the Fc region—interacts with receptors on immune effector cells. Structurally speaking, immunoglobulins are heterodimeric proteins composed of two ~55 kDa polypeptide chains dubbed the “heavy” chains (H) and two ~25 kDa polypeptide chains dubbed the “light” chains (L). Based on the specific composition of the heavy chains, immunoglobulins can be categorized into a number of different isotypes, including IgA, IgM, IgD, IgE, and IgG. In contrast, there are only two types of light chains: kappa (κ) and lambda (λ). For the sake of simplicity, we will limit this discussion of antibody structure to IgG molecules, the most abundant isotype in antiserum.

The heavy and light chains are composed of a number of segmented domains, which are broadly categorized as the *constant* (C) and *variable* (V) domains. Each domain has 110–130 amino acid residues, averaging a molecular weight of 12.5 kDa [32]. While the heavy chain of a typical IgG has three C domains (C_H_1, C_H_2, C_H_3) and one V domain (V_H_), the light chain is made up of one V domain (V_L_) and one C domain (C_L_). Taken together, there are a total of 12 individual domains per IgG molecule. These domains are organized further into a three-dimensional structure which is primarily held together by noncovalent hydrophobic interactions, hydrogen bonds, and van der Waals forces. However, covalent disulfide bonds play a very important role in the structure of IgGs as well. Typically, IgGs possess 16 disulfide bonds formed between 32 cysteine residues. Four of these linkages are interchain disulfide bonds: two in the flexible hinge region and two that connect the constant domains within the Fab region: C_H_1 with C_L_. The remaining 12 are intrachain disulfide bonds, with one linkage per domain. Considering the topic at hand, it is important to note that the abundance of hydrophobic interactions between the various domains allows for the inter- and intrachain disulfides to be partially reduced or even substituted without compromising the structural integrity of the antibody [33]. The key to the extraordinary specificity of antibodies lies in the V domains of the immunoglobulin. The V domains of the Fab region are composed of four framework regions interspersed with three hypervariable complementarity determining regions: CDR1, CDR2, and CDR3 [34, 35]. The framework regions principally contribute to the stability and interdomain interactions between the heavy and light chain domains. The unique antigen-binding pocket, or paratope, is created by the three-dimensional organization of the CDRs of both the L and H chains and ultimately confers diversity and specificity to an antibody for its target antigen [10].

The posttranslational glycosylation of antibodies adds an additional structural element and has important implications for their function as well [36]. Antibodies are glycoproteins, and different isotypes are characterized by different degrees of glycosylation. IgGs, for example, are known to have an overall 3 % carbohydrate content, with a conserved glycosylation site at N297 on both C_H_2 domains within the Fc region. Glycans attached to this residue comprise a complex biantennary heptasaccharide unit formed by D-galactose (Gal), *N*-acetyl-d-galactosamine (GalNAc), *N*-acetylglucosamine (GlcNAc), l-fucose (Fuc), and d-mannose (Man). In addition to contributing to the proper folding and solubility of immunoglobulins, glycans impact the downstream activation of immune effector functions by virtue of their interaction with the complementary Fc receptors on immune effector cells [37].

### Antibody Fragments

As early as 1950, experiments by Porter *et al.* found that antibodies can be digested with enzymes to produce two independent Fab fragments and an Fc fragment [38–40]. Not long after, it was found that peptic digestion of antibodies yielded two products: a dimeric F(ab′)_2_ and an Fc fragment [41]. Purification of these moieties revealed that the isolated Fab and F(ab′)_2_ units were capable of binding to the target antigen of the parent antibody with specificity and selectivity, albeit with different valencies. This work has led to the emergence of antibody engineering and the production of a variety of antibody fragments based on the smallest, completely functional monovalent antigen-binding unit of an IgG: the single-chain variable fragment (scFv) (~28 kDa) [28, 42].

From an imaging point of view, antibody fragments offer a number of enticing traits, including (a) rapid clearance from systemic circulation, (b) better extravasation and tumor penetration than full-length antibodies, and (c) immunologic inertness due to the absence of the Fc region. Furthermore, the recombinant technology used to produce these fragments provides an opportunity to introduce genetic modifications to improve target avidity and binding valence as well as to facilitate bioconjugation [28]. Indeed, beyond F(ab′)_2_, Fab, and scFv fragments, a variety of other engineered constructs have been created, including diabodies (Db), cys-diabodies (cysDb), minibodies (Mb), single-domain antibodies (sdAb), and scFv-Fc fusion constructs (Fig. 1) [28, 42–44]. Immunoconjugates based on these fragments have demonstrated significant promise in preclinical imaging investigations; admittedly, however, the clinical potential of engineered fragments has yet to be fully realized.

## Cysteine Residues

**Fig. 4:**
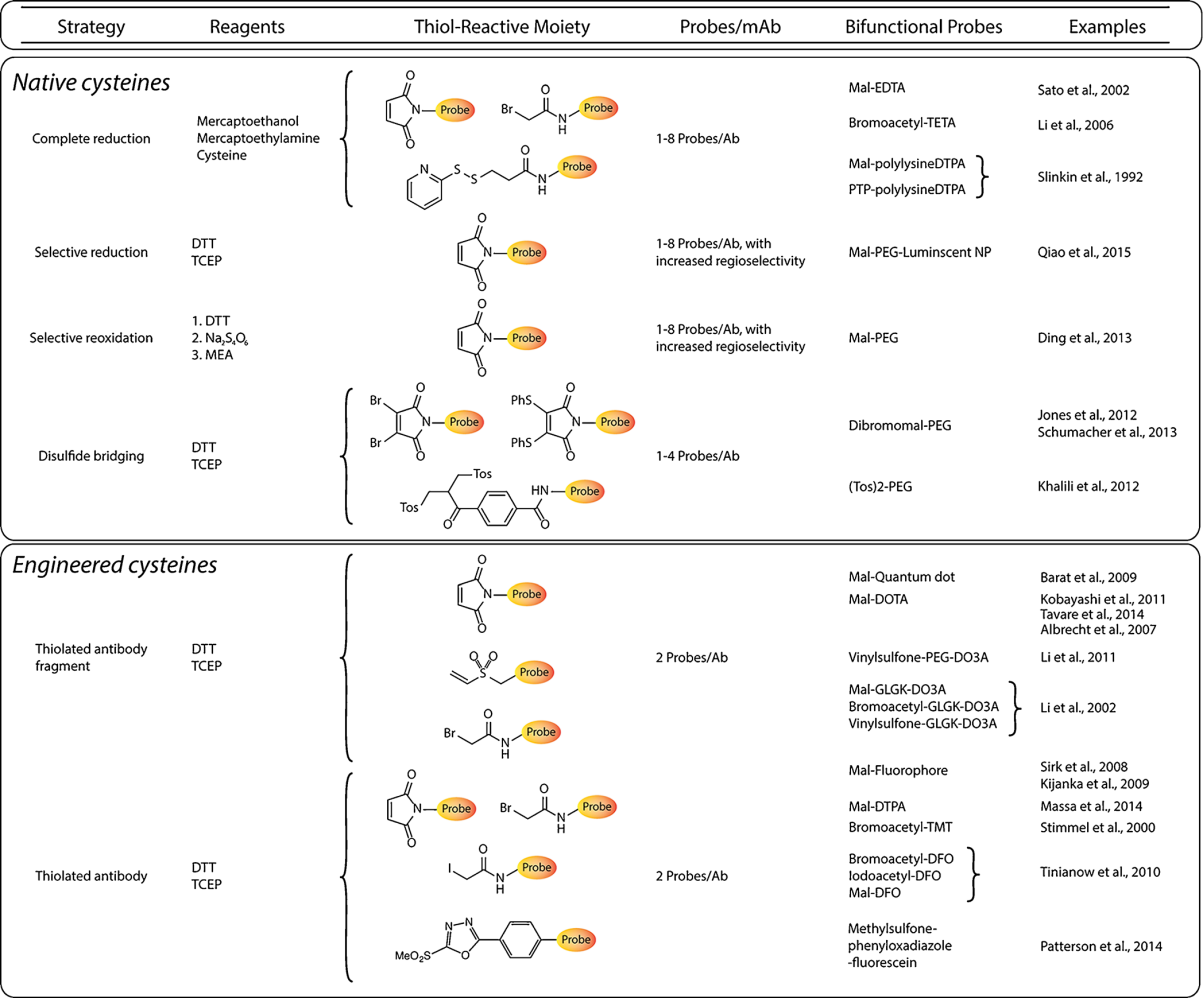
Table of site-specific bioconjugation strategies based on the modification of cysteine residues.

Cysteine residues and their thiol functional groups have long been attractive targets for the selective modification of peptides and proteins [45]. Much like the lysines targeted in traditional bioconjugation approaches, cysteine residues occur naturally within antibodies; importantly, however, antibodies contain fewer cysteines than lysines, and these cysteine residues occur only at specific and well-defined locations within the immunoglobulin. From a bioconjugation standpoint, the most enticing trait of cysteines is their ability to undergo highly selective ligations *via* Michael additions and alkylations. The most commonly employed thiol-reactive moiety is the maleimide, which undergoes a Michael addition with the sulfhydryl group to form a maleimidyl-thioether bond (Fig. 2c). However, many have argued that this linkage is less than ideal for bioconjugation due its instability to hydrolysis and propensity for exchange reactions with endogenous, thiol-bearing proteins. As a result, significant effort has been dedicated to the development of more efficient thiol-reactive constructs (Fig. 2d–h) [22, 46, 47]. As we have discussed, full-length IgGs typically contain 32 cysteine residues that combine to form 12 *intra*chain and 4 *inter*chain disulfide bridges. Naturally, these numbers go down when considering antibody fragments: Fab fragments, for example, possess four intrachain and one interchain disulfide bridges. Interchain disulfides are the more attractive natural conjugation targets, both because they are more easily reduced than their intrachain counterparts and because of their position far from the antigen-binding domains. However, some laboratories have sought to move past the modification of naturally occurring disulfides, instead using genetic engineering to incorporate free cysteine residues into immunoglobulins with the express purpose of creating conjugation sites (Fig. 4). In this section, we will discuss approaches that have been developed to site-specifically modify full-length IgGs and smaller fragments using both native and engineered cysteine residues.

### Native Cysteines

Without a doubt, the simplest thiol-based site-specific modifications are those made to native cysteines. For example, in 2002, Sato *et al.* created an anti-tenascin-C (TNC) Fab′ *via* the digestion of an anti-TNC IgG with pepsin and the subsequent reduction of the disulfides in the hinge region [48]. The free sulfhydryl groups were then conjugated to a maleimide-bearing variant of ethylenediaminetetraacetic acid (EDTA), and the unreacted thiols were quenched with iodoacetamide, yielding a conjugate with a degree of labeling of 1.4 EDTA/Fab′ (Fig. 3). After radiolabeling the Fab′-EDTA construct with In-111, the radioimmunoconjugate was successfully employed for *in vivo* imaging in a murine model of myocarditis. Using a very similar strategy, another laboratory created a CA125-targeting Fab′ fragment from the mouse IgG B43 [49]. In this case, however, instead of a maleimide-bearing construct, the researchers employed a bifunctional variant of 1,4,8,11-tetraazacyclotetradecane-1,4,8,11-tetraacetic acid (TETA) (Fig. 3) with a pendant bromoacetamide group (BAT) for conjugation to the free, hinge region thiols. The TETA-modified Fab′ was then radiolabeled with Cu-67, and it was determined that the completed radioimmunoconjugate possessed immunoreactivity comparable to the unmodified Fab′. However, no reports of *in vivo* experimentation with the [^67^Cu]TETA-Fab′ construct could be found in the initial report or any follow-up publications. In a third example, over 20 years ago, Slinkin *et al.* created an anti-carcinoembryonic antigen (CEA) Fab′ *via* the digestion of a full-length IgG with pepsin and the reduction of the hinge region disulfides of the F(ab′)_2_ intermediate with mercaptoethylamine (MEA) [50]. The authors then “activated” the Fab′ using Ellman’s reagent (DTNB), isolated the reactive Fab′-TNB, and reacted the fragment with two different diethylenetriaminepentaacetic acid (DTPA)-bearing polylysine constructs: one bearing a maleimide to create a thioether linkage and another containing a (pyridyldithio)proprionate functionality geared toward the production of a reducible disulfide bridge. After radiolabeling the immunoconjugates with In-111, a biodistribution study was carried out in mice bearing LS174T human colorectal carcinoma xenografts. Interestingly, while the thioether-linked radiotracer was shown to target the tumor efficiently, the disulfide-bridged compound resulted in high levels of kidney uptake and poor tumor targeting, likely the result of the *in vivo* cleavage of the S-S linkage between the radiometal and the antibody.

While the methods described above work very well for the modification of Fab′ fragments, they are not appropriate for intact antibodies. After all, IgGs contain four *inter*chain disulfide bridges, and their nonspecific reduction can create as many as eight different free cysteines [51]. Needless to say, the modification of an antibody bearing eight different conjugation sites hardly qualifies as site-specific. In order to circumvent this issue, a number of laboratories have employed strategies geared toward the selective reduction of disulfide linkages [52]. In an excellent example, Sun *et al.* explored these possibilities during their efforts to site-specifically conjugate a maleimide-bearing variant of monomethyl auristatin E (MMAE) to an anti-CD30 mAb [53]. The authors report that the reducing agents dithiothreitol (DTT) and tris(2-carboxyethyl)phosphine (TCEP) can preferentially reduce the disulfide bonds bridging the heavy and light chains when used in small amounts. Interestingly, it was also found that the *same* disulfide bridges can be preferentially oxidized when the fully reduced IgG is subjected to reoxidation. Armed with this information, the authors were able to create a variety of ADCs and test their performance *in vitro* and *in vivo*. It is important to note that the isomeric homogeneity of these constructs ranged from 60 to 90 %, values that are impressive yet certainly leave room for improvement.

Most of the strategies discussed above are accompanied by the loss of interchain disulfide bridges. While this is generally tolerated, it is far from ideal, as these interchain links confer stability to the antibodies. Two different groups in the UK have circumvented this issue by employing bifunctional constructs that are capable of attaching a cargo to the immunoglobulin while also establishing a covalent interchain link. In one case, this was achieved through the use of a polyethyleneglycol (PEG)-modified dibromomaleimide moiety capable of performing two separate nucleophilic substitutions with the cysteines that had once formed an interchain disulfide bond (Fig. 2g) [54]. In the other example, the authors employed a PEG-containing bisulfone group that is likewise capable of covalently relinking the erstwhile disulfide thiols [55]. Finally, Schumacher *et al.* have also used divalent maleimides—specifically dibromomaleimides and dithiophenolmaleimides—to functionalize antibody fragments while retaining a covalent crosslink where a disulfide once existed (Fig. 2g–h) [56].

### Engineered Cysteines

An alternative to the use of native cysteine residues lies in the genetic incorporation of engineered cysteines as bespoke modification sites. This approach comes with both advantages and disadvantages. On the plus side, it allows the native cysteine residues of the immunoglobulin to remain intact, thereby eliminating the possibility of any harm to the antibody. In addition, the use of engineered thiol sites allows the researcher to precisely tailor both the location and number of conjugation sites. On the other hand, it has been shown that free, unpaired cysteine residues can spontaneously oxidize to form undesired disulfide bridges, leading to aggregation and structural modifications [57, 58]. Moreover, the location of the incorporation site must be chosen very carefully in order to eliminate the risk of interfering with the antigen-binding domains. In response to these issues, Junutula *et al.* developed the phage ELISA for selection of reactive thiols (PHESELECTOR) biochemical assay, a procedure that provides information on the influence that the site of the introduced cysteine has on antigen-binding affinity *as well as* the ability to covalently modify the thiol in question [59]. Finally, the genetic engineering of immunoglobulins undeniably adds complexity and expense to the synthetic process as well. This is particularly true for full-length IgGs and F(ab′)_2_ fragments. However, even the most basic syntheses of diabodies, minibodies, or scFv require genetic engineering. Thus, in these cases, the genetic incorporation of additional cysteine residues can be achieved with relatively little added effort.

In the last few years, a number of laboratories have employed genetic engineering to create site-specifically modified immunoconjugates based on antibody fragments. In 2014, for example, Anna Wu’s laboratory reported the development of anti-activated leukocyte cell adhesion molecule (ALCAM) cysDb [60]. After reduction with TCEP, these constructs were conjugated to a maleimide-bearing bifunctional 1,4,7,10-tetraazacyclododecane-1,4,7,10-tetraacetic acid (DOTA) to yield constructs with exactly two chelators per diabody (Fig. 3). For the sake of comparison, the cysDbs were also randomly modified using DOTA-NHS. Both immunoconjugates were successfully radiolabeled with ^64^Cu and used for PET imaging in mice bearing ALCAM-positive and ALCAM-negative xenografts. While both Cu-64-labeled diabodies effectively discriminated between the two tumor types, the site-specifically labeled [^64^Cu]DOTA-cysDb exhibited higher tumoral uptake and more favorable tumor-to-background activity concentration ratios than its randomly labeled cousin. In addition, the site-specific labeling of the cysDb also seemed to exert a significant influence on the pharmacokinetic profile of the radioimmunoconjugate, increasing kidney uptake and decreasing liver retention compared to the randomly labeled construct.

**Fig. 5:**
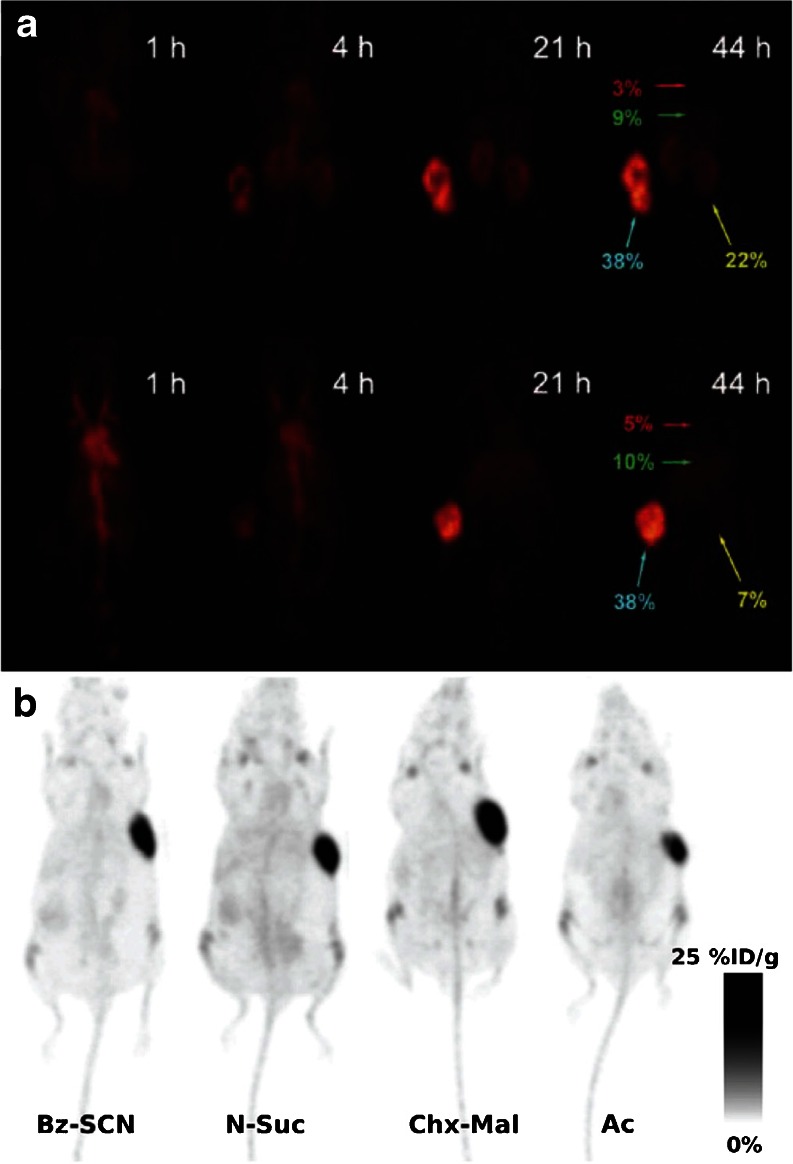
**a** Serial PET images of site-specifically labeled [^64^Cu]DOTA-PEG_24_-AVP04-50 (*top*) and [^64^Cu]DOTA-PEG_48_-AVP04-50 (*bottom*) in athymic nude mice bearing LS174T xenografts. The labels in *red*, *green*, *yellow*, and *turquoise* illustrate the %ID/g values in the heart, liver, kidney, and tumor, respectively. Figure adapted and reprinted with the permission of Li *et al.* Copyright 2011 American Chemical Society [65]. **b** PET images of four different variants of [^89^Zr]DFO-thio-trastuzumab in mice bearing BT474 xenografts. In two of the radioimmunoconjugates, the chelator was attached using nonsite-specific conjugation methods (Bz-SCN and *N*-Suc), while in the other two constructs, bioconjugation was achieved using thiol-reactive variants of DFO (Chx-Mal and Ac). Figure adapted and reprinted with the permission of Tinianow *et al.* Copyright 2010 Elsevier Publishing Group [17].

Other laboratories have synthesized a range of site-specifically radiolabeled fragments *via* reactions with C-terminal cysteines, including an anti-TNC [^111^In]DTPA-scFv, an anti-human epidermal growth factor receptor 2 (HER2) [^111^In]DTPA-sdAb, an anti-MUC1 [^111^In]DOTA-di-scFv, and an anti-CEA [^64^Cu]DO3A-GLGK-cysDb (Fig. 3) [61–64]. In a small variation on this approach, Li *et al*. employed DO3A-PEG_n_ constructs (*n* = 12, 24, and 48) bearing a vinyl sulfone moiety to site-specifically modify an anti-TAG-72 diabody with a C-terminal cysteine (Fig. 3 and 5a) [65]. After radiolabeling these conjugates with Cu-64, PET imaging was performed using mice bearing LS174T xenografts, and the authors found that while all of the diabodies proved able to target the tumor, the background activity levels in the blood and kidneys were highly dependent on the length of the PEG chain. Conjugations with C-terminal cysteines have also been used for the creation of immunoconjugates for fluorescence imaging. For example, Sirk *et al.* used maleimide-bearing variants of AlexaFluor® 488, phycoerythrin, and allophycocyanin to create anti-HER2 and anti-CD20 cysDbs for *in vitro* fluorescence imaging (Fig. 3) [66]. More recently, a group in the Netherlands reported the development of a series of HER2-targeting nanobodies site-specifically conjugated to the near-infrared fluorophore IRDye 800CW through C-terminal cysteine residues (Fig. 3) [67]. *In vivo* fluorescence imaging experiments using mice bearing HER2-positive SKBR3 breast cancer xenografts revealed that the site-specifically labeled nanobodies yielded higher tumor-to-background intensity ratios than a construct that had been randomly modified through lysine residues. Moreover, these probes were shown to possess significant promise for intraoperative imaging during the surgical resection of tumors.

In their efforts to reduce the retention of radioisotopes in the kidneys—a frequent stumbling block for radiolabeled antibody fragments—the laboratories of Wu and Shively provided an excellent comparative case study on the various thiol-reactive conjugation strategies [68]. In this work, the authors incorporated a GLGK tetrapeptide linker between an anti-CEA cysDb and a DO3A chelator that is designed to be cleaved specifically by the carboxypeptidase activity of kidney brush border enzymes (Fig. 3). In theory, this modification could facilitate the specific cleavage of the tetrapeptide upon the inevitable uptake of the fragment in the kidney, thereby facilitating the rapid elimination of the radiometal-chelate complex even though the diabody remains trapped in the kidney. To this end, GLGK peptides were functionalized on the N-terminus with a DO3A chelator and on the ε amino group of the lysine residue with three different thiol-reactive moieties: a maleimide, a bromoacetyl group, and a vinylsulfone group. These DO3A-peptide constructs were then site-specifically attached to the C-terminal cysteine of the diabody, producing conjugates with degrees of labeling ranging from 0.8 to 1.3 chelators/cysDb. These constructs—along with a nonsite-specifically labeled variant—were labeled with In-111, and biodistribution experiments were performed in mice bearing LS174T colon cancer xenografts. As expected, high activity concentration levels were observed in the kidneys for the randomly modified conjugate [^111^In]DOTA-cysDb but also, somewhat surprisingly, the bromoacetyl-based [^111^In]DO3A-GLGK-cysDb construct. In contrast, dramatically reduced kidney uptake was observed for the conjugates created using the maleimide and vinylsulfone approaches. This clearly underscores that the conjugation strategy may have as much of a role in influencing biodistribution as the addition of the cleavable peptide.

The incorporation of cysteines *via* genetic engineering has also been applied to full-length IgGs to produce what has often been dubbed “thiomAbs.” Stimmel *et al.*, for example, mutated position 442 in the C_H_3 domain of an IgG to replace a serine residue with a cysteine [69]. The mutant IgG was then partially reduced with MEA to free the engineered thiol while leaving the native disulfides untouched. This construct was then conjugated to a bromoacetamide-bearing variant of the TMT chelator to yield a final construct with between one to two chelators/mAb and an immunoreactivity nearly identical to that of the unmodified antibody (Fig. 3). Much more recently, a team from Genentech developed a genetically engineered variant of trastuzumab with cysteine residues incorporated in the heavy chain [17]. This thiomAb was reduced with an excess of DTT and then partially reoxidized with dehydroascorbic acid in order to selectively prepare the engineered thiols for modification with variants of the Zr-89 chelator desferrioxamine (DFO; Fig. 3) bearing thiol-reactive bromoacetyl, iodoacetyl, or maleimide groups (Fig. 5b). These trastuzumab-DFO immunoconjugates were found to have approximately 1.8 DFO/mAb, were labeled with Zr-89 in high yield and radiochemical purity, and were found to have immunoreactivities and stabilities comparable to constructs created using nonsite-specific conjugation methodologies. In a separate study, Boswell *et al.* developed a maleimide-bearing tyrosine-DOTA construct as a scaffold for the site-specific iodination of immunoconjugates. The tyrosine-DOTA moiety was first labeled with I-125 and subsequently attached to a HER2-targeting thioMab, with the ultimate aim of developing residualizing radioiodinated antibodies for both PET imaging (using I-124) and therapy (using I-131) [70].

ThiomAbs can also be enzymatically digested to afford F(ab′)_2_, Fab′, and Fab fragments bearing engineered cysteine residues. Using this approach, the same group at Genentech created a HER2-targeting thioFab [71]. This fragment was then conjugated to an F-18-labeled PEGylated maleimide moiety that had been prepared *via* copper-catalyzed azide-alkyne click chemistry (Fig. 2i). In order to explore alternate synthetic strategies, the same radioimmunoconjugate was also synthesized in a two-step procedure based on the initial bioconjugation of an alkyne-bearing PEGylated maleimide followed by the copper-catalyzed ligation of an azide-containing F-18-labeled synthon. However, the authors ultimately concluded that the former strategy is preferable, as it precludes any degradation of the antibody fragment by the Cu^I^ click chemistry catalyst.

All of the cysteine-based modification strategies we have discussed offer enticing possibilities. The approaches based on the manipulation of native cysteines are refreshingly simple and require no genetic engineering, while the methods employing engineered cysteines offer unprecedented levels of regiochemical control. However, a major limitation to *all* these strategies lies in the suboptimal biological stability of maleimdyl thioether bonds. Other thiol-reactive constructs have been used effectively, yet the maleimide-thiol Michael addition reaction persists as the standard technology for cysteine-based conjugations. We are confident, however, that the next few years will witness increases in the use of more suitable chemical tools for thiol-based conjugations, such as phenyloxadiazole sulfones, dibromomaleimides, and dithiophenolmaleimides (Fig. 2g–h, j) [22, 46, 47].

## Glycans

**Fig. 6:**
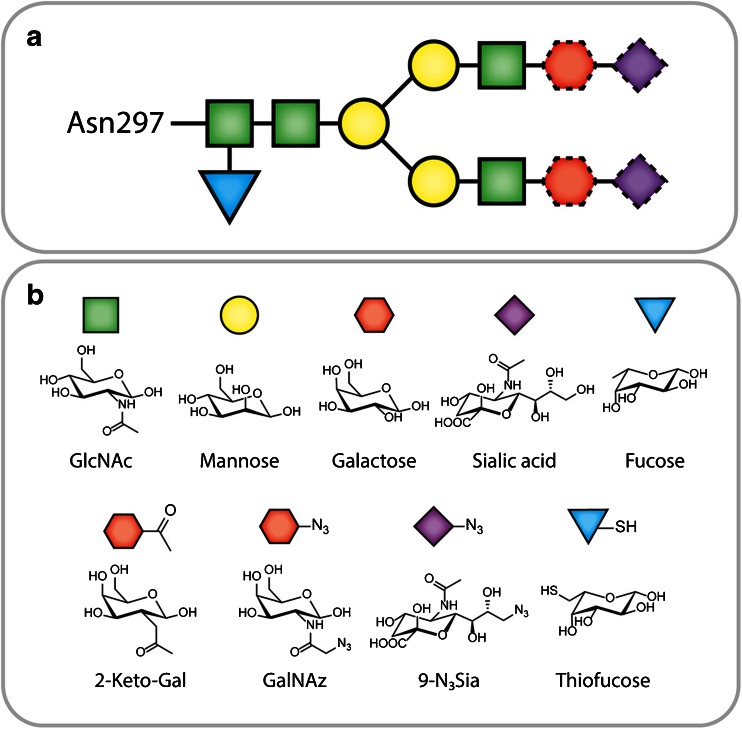
**a** The biantennary structure of the heavy chain glycans; the *dotted* outlines indicate residues that are not *always* present in the glycans. **b** Structures of natural and synthetic monosaccharides.

IgGs contain two conserved glycosylation sites—the N297 residues in the C_H_2 domains of the heavy chains—each bearing a biantennary, complex-type oligosaccharide chain (Fig. 6a). These heavy chain glycans have three significant advantages as a platform for site-specific modification: (1) the heavy chain C_H_2 domains lie far from the antigen-binding regions of the IgG, thus minimizing the risk of inadvertently impairing the immunoreactivity of the antibody; (2) the basic chemistry of sugars differs fundamentally from that of amino acids, meaning that the glycans can be manipulated without disturbing the polypeptide chain; and (3) the biantennary nature of the two oligosaccharide chains opens the door for at least two and as many as four conjugation events per antibody.

### Oxidation-Based Methods

**Fig. 7:**
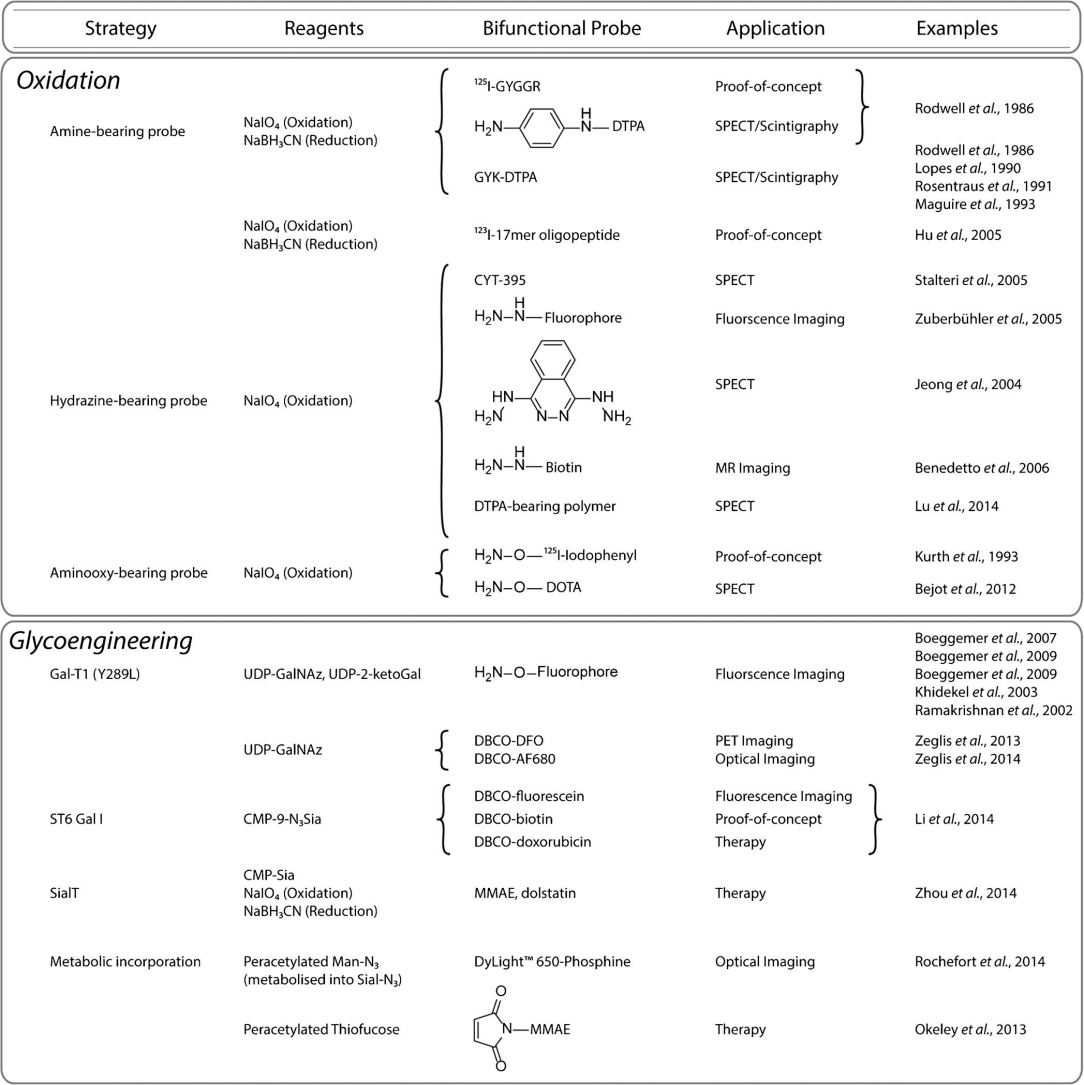
Site-specific bioconjugation strategies based on the modification of glycans.

The oldest methodologies for the site-specific modification of antibodies rely on the oxidation of the heavy chain glycans (Fig. 7) [8, 16, 72–74]. It is well known that sugars can be oxidized using periodate (IO_4_^−^) to create aldehydes (Fig. 2k). These aldehydes can then react selectively with nucleophiles—including amines, hydrazide, and aminooxy groups—to form covalent linkages (Fig. 2l–p). Importantly, there are two major caveats to these oxidation-based conjugation methodologies. First, some of the bonds formed *via* the reaction of the aldehydes with nucleophiles—for example, imine linkages—are hydrolytically unstable, and therefore, a reduction step is required to obtain stable immunoconjugates. Second, the initial treatment of the glycans with periodate is not always benign: it is known that this step may also result in the oxidation of methionine residues, which may in turn inadvertently affect the ability of the antibody to bind its antigen [75].

In 1986, Rodwell *et al.* first applied this method to the synthesis of site-specifically modified radioimmunoconjugates [26, 76]. In this work, the major histocompatibility complex (MHC)-targeting antibody R9.75 was oxidized using NaIO_4_, coupled to one of three moieties bearing a primary amine—an I-125 labeled pentapeptide ([^125^I]GYGGR), a DTPA-bearing tripeptide (GYK-DTPA), or a *p*-aminoaniline-DTPA—and finally reduced using sodium cyanoborohydride (Fig. 2l). After radiolabeling with In-111, the immunoconjugates were successfully used to image mice bearing lymphoma xenografts *via* scintigraphy. A comparison with nonsite-specifically modified immunoconjugates illustrated that the site-specifically labeled radioimmunoconjugates targeted tumor tissue far more effectively. In more recent years, other groups have followed similar strategies for the synthesis of radioiodine-labeled constructs [74]. In addition, a number of site-specifically modified [^111^In]DTPA-labeled radioimmunoconjugates have been made using the chelator GYK-DTPA, including examples based on the anti-prostate-specific membrane antigen (PSMA) antibody 7E11-C5, the anti-carcinoembryonic antibody C46, the breast cancer-targeting murine IgG 15A8, and the TAG72-targeted antibody B72.3 (*i.e.*, satumomab) [77–80]. All of these radioimmunoconjugates have been shown to successfully target tumor tissue in murine models of cancer, and notably, [^111^In]DTPA-satumomab has been used in the clinic in patients with colorectal cancer [80].

The aldehydes produced by the oxidation of the glycans can also react with hydrazides to form hydrazone-based linkages (Fig. 2m). While hydrazones possess greater innate stability than their imine cousins, hydrazones can also be reduced *via* sodium cyanoborohydride to create hydrazine-based linkages, further increasing their durability [81]. Along these lines, Stalteri *et al.* oxidized and coupled the PSMA-targeting antibody 7E11C5.3 with the hydrazine-bearing chelator CYT-395, radiolabeled the resulting immunoconjugate with Tc-99m, and were able to effectively image prostate cancer tumors in patients (Fig. 3) [82, 83]. Using similar methods, Zuberbühler *et al.* employed hydrazide-bearing fluorophores to create a fluorescent immunoconjugate based on the anti-fibronectin antibody F8 [84]. Another group took a slightly different approach, modifying the anti-CD5 antibody T101 with dihydrazinophthalazine (DHZ), a compound bearing two hydrazides: while one reacted with the aldehyde to site-specifically modify the antibody, the other was subsequently employed as part of a coordination scaffold for Tc-99m (Fig. 2n*)* [85]. The authors found that the resulting site-specifically Tc-99m-labeled radioimmunoconjugate proved more stable than an analogous, traditionally conjugated variant. More recently, in an effort to develop In-111-labeled immunoconjugates for Auger electron radiotherapy, Lu *et al.* site-specifically conjugated polymers bearing ~30 DTPA each to trastuzumab *via* oxidation with NaIO_4_, reaction with the hydrazide-bearing polymers, and reduction with NaBH_3_CN [86]. The resulting constructs were shown to have approximately 1.2 polymers/mAb and could be successfully labeled with ^111^In in high yield and at higher specific activities than traditional, lysine-conjugated trastuzumab-DTPA. The authors illustrated that the modification of the In-111-labeled radioimmunoconjugate did not have a deleterious effect on the *K*_D_ of the antibody for HER2; however, the polymer-modified antibodies showed significantly increased nonspecific binding to cells that did not express the target antigen. While a follow-up study published in 2015 presented some promising *in vitro* results, no *in vivo* data was provided in either report, leaving the pharmacokinetic influence of the DTPA-laden polymers unknown for now [87].

A final variation on this theme employs O-alkyl hydroxylamines as the nucleophile in order to form aldehyde oxime ethers, which are more hydrolytically stable than imine or hydrazone products and do not require a subsequent reduction step (Fig. 2o–p) [88]. Kurth and colleagues applied this strategy to the development of a radioimmunotherapeutic agent, using an aminooxy-bearing, I-125-labeled iodophenyl construct and the mAb 35, which targets the Gold 3 epitope of the carcinoembryonic antigen [89]. The site-specifically labeled I-125 mAb 35 radioimmunoconjugates were synthesized in high specific activity and immunoreactivity and were shown to be highly stable. More importantly, in biodistribution experiments using mice bearing subcutaneous T380 colorectal cancer xenografts, the site-specifically labeled radioimmunoconjugate was found to have higher tumor retention and lower thyroid uptake than a variant produced using a nonsite-specific radioiodination method. Unfortunately, however, we were unable to find any *in vivo* therapy data using this construct or, perhaps more appropriately, a I-131-bearing analog. More recently, Bejot *et al.* followed a similar route to label trastuzumab with an aminooxy-bearing variant of DOTA, producing a site-specifically labeled radioimmunoconjugate with 5.1 ± 0.7 DOTA/mAb, a very high immunoreactive fraction, and low nonspecific binding to HER2-negative cells. Subsequent SPECT imaging experiments using mice bearing subcutaneous, bilateral MDA-MB-361 (HER2-positive) and MDA-MB-231 (HER2-negative) breast cancer xenografts illustrated that the site-specifically labeled [^111^In]DOTA-trastuzumab specifically targeted the HER2-expressing xenografts but did not offer a significant improvement over an [^111^In]DOTA-trastuzumab construct synthesized using a traditional, nonsite-specific conjugation method [90].

### Glycoengineering Methods

Over the last 15 years, a number of alternative chemoenzymatic methods for the specific functionalization of glycoproteins have emerged. Using both natural and engineered enzymes, it is now possible to introduce carefully tailored sugars into the glycans to enable chemoselective modifications. Not surprisingly, this work has been enthusiastically applied to the creation of site-specifically labeled radioimmunoconjugates.

The most well-known enzyme used in these methodologies is Gal-T1(Y289L), a mutant β-1,4-galactosyltransferase developed by Qasba and coworkers. This substrate-permissive galactosyltransferase facilitates the attachment of modified galactose monomers to *N*-acetylglucosamine (GlcNAc) residues in the glycans (Fig. 6b) [91]. Two modified galactose residues have served as focal points: 2-acetyl-2-deoxy-galactose (2-keto-Gal) and *N*-azido-acetylgalactosamine (GalNAz; Fig. 6b). Each of these monomers can be used for bioorthogonal conjugations: the former can be reacted with nucleophiles in a manner similar to oxidized sugars while the latter is obviously a substrate for a variety of click chemistry transformations (Fig. 2i). Both of these unnatural sugars have been successfully incorporated into antibodies using Gal-T1(Y289L) to demonstrate proof-of-concept and to create fluorescently labeled antibodies for *in vitro* imaging applications [92, 93].

**Fig. 8:**
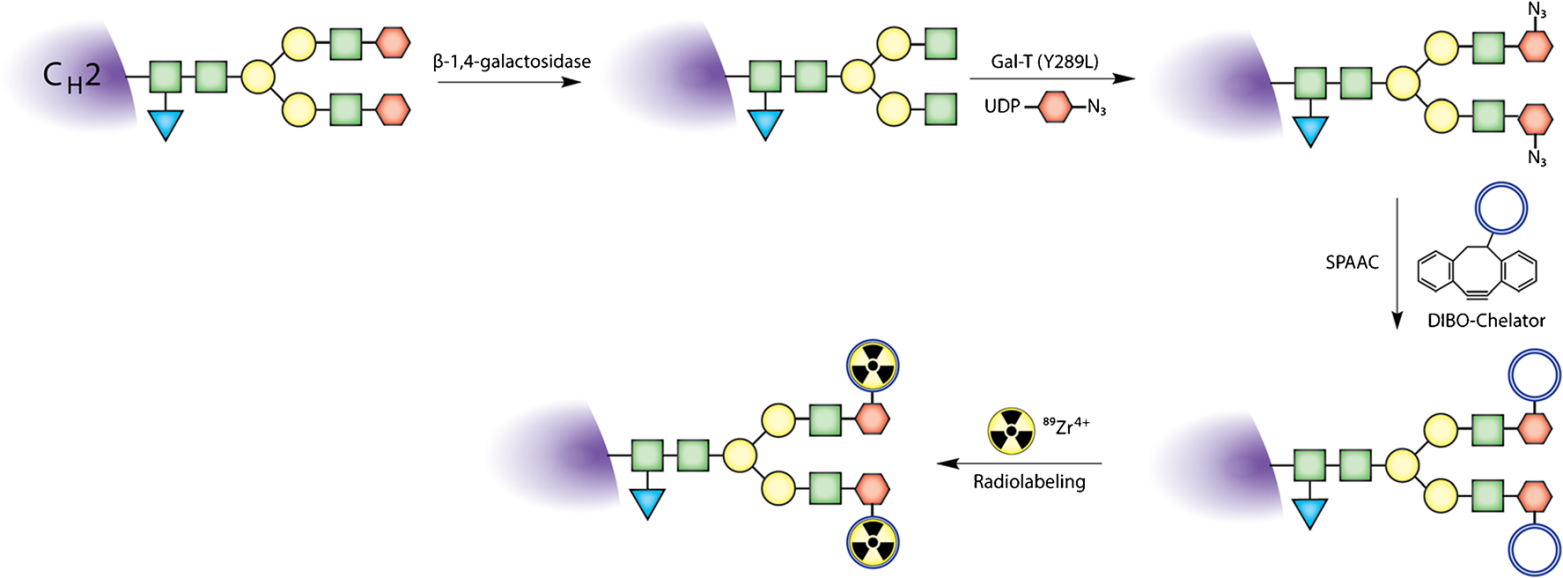
Schematic of a Gal-T(Y289L)-based site-specific modification procedure.

**Fig. 9:**
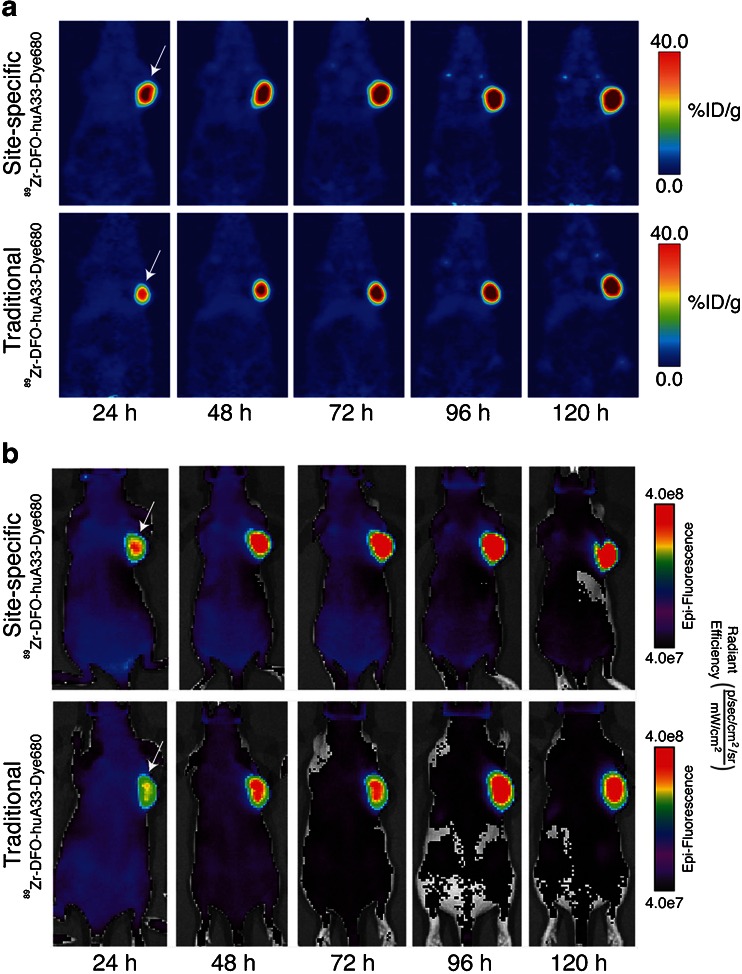
**a** PET and **b** near-infrared fluorescence images of athymic nude mice bearing SW1222 tumors (*white arrows*) injected with either site-specifically labeled or traditionally labeled [^89^Zr]DFO-huA33-Alexa Fluor® 680. In the PET images, the coronal slices intersect the center of the tumors. Figure adapted and reprinted with the permission of Zeglis *et al.* Copyright 2014 American Chemical Society [18].

The first application of this Gal-T1(Y289L)-based methodology for the construction to nuclear imaging agents was published in 2013 [13]. In this work, Zeglis *et al.* employed a three-step modification procedure: (1) the removal of terminal galactose residues of the glycans using β-1,4-galactosidase, (2) the attachment of Gal-NAz to the sugar chains using Gal-T1(Y289L), and (3) the conjugation of chelator-modified dibenzocyclooctynes to the glycans *via* the strain-promoted azide-alkyne click reaction (Figs. 2q and 8). Using this methodology, the authors created a desferrioxamine (DFO)-bearing immunoconjugate of the PSMA-targeting antibody J591 and subsequently labeled this construct with Zr-89. *In vivo* PET imaging and biodistribution experiments in mice bearing subcutaneous PSMA-expressing LNCaP prostate cancer xenografts suggested that the site-specifically labeled radioimmunoconjugate produces slightly higher absolute tumoral uptake (67.6 ± 5.0 %ID/g at 96 h postinjection) than an analogous agent produced using a traditional, nonsite-specific modification strategy (57.5 ± 5.3 %ID/g at 96 h postinjection). In a subsequent study, the same group used an improved, one-pot modification strategy to create a series of DFO- and AlexaFluor® 680-bearing immunoconjugates based on the huA33 antibody for the multimodal PET/near-infrared fluorescence imaging of colorectal cancer (Fig. 9) [18]. In this work, PET and fluorescence imaging experiments suggested that the site-specifically modified radioimmunoconjugates exhibited comparable—if not slightly superior—*in vivo* behavior compared to variants produced using non-site-specific conjugation methods.

In an effort to create more homogeneously functionalized glycans chains, Boons and coworkers have recently developed a modified chemoenzymatic strategy [94]. In this strategy, terminal galactose residues are first added to the glycans using galactosyltransferase (GalT) and uridine diphosphate (UDP)-galactose. Then, an azide-modified sialic acid monomer is incorporated into the glycans chain using sialyltransferase (ST6GalI) and the donor substrate CMP-9-N_3_Sia. Finally, strain-promoted azide-alkyne click chemistry is employed to attach dibenzocyclooctyne (DBCO)-modified cargoes to the antibody. This glycans remodeling strategy yielded 3.5 and 4.1 drugs/mAb for the control and anti-CD22 antibodies, respectively, strongly suggesting quantitative labeling of the termini of the heavy chain glycans. Using a similar strategy, Zhou *et al.* sought to increase the amount of sialic acid in the glycan chains *via* modification with GalT and then sialyltransferase (SialT) [12]. These sialic acid monomers were then specifically oxidized under mild conditions, and the resulting aldehydes were used to conjugate aminooxy-bearing variants of MMAE (Fig. 3) and dolastatin 10 (Dol10; Fig. 3).

A more elegant method for the incorporation of orthogonally functionalized sugars into glycoproteins lies in harnessing the metabolism of cells. This method consists of enriching the media of cells with modified acetyl-bearing sugars and relying on the cells themselves to incorporate these monomers into glycoproteins. In 2014, Rochefort *et al.* used this method to prepare an anti-CA19-9 antibody site-specifically modified with azide groups *via* the metabolic incorporation of peracetylated *N*-azido-acetylmannosamine [95]. After purification, the azide-modified antibody was then labeled with a phosphine-bearing fluorophore (DyLight-650) *via* the Staudinger ligation, and *in vivo* fluorescence imaging was used to show that the immunoconjugate was specifically taken up in BxPC3 pancreatic adenocarcinoma xenografts (Fig. 2r). The primary drawback of this method, however, is the poor incorporation of functionalized sugars: a fluorophore/mAb ratio of only 1:11 was achieved. Okeley and coworkers used a similar metabolic engineering approach to incorporate 6-thiofucose site-specifically into the heavy chain glycans of the CD30-targeting antibody cAC10 and the CD70-targeting antibody h1F6 (Fig. 6b) [96, 97]. The authors found that 1.2–1.4 thiofucose monomers were incorporated per antibody and—using a cysteine reduction/reoxidation strategy and a maleimide-bearing variant of MMAE—created immunoconjugates bearing 1.3 drugs/mAb that proved more stable to decomposition *via* retro-Michael addition than an analogous immunoconjugate in which the interchain disulfides had been modified.

As the recency of these citations illustrates, the use of metabolic glycans engineering to create site-specifically modified antibodies—let alone site-specifically modified antibodies for imaging applications—remains a very young field. Thus, in order to maximize the benefits of this technology, it is crucial that imaging-focused laboratories continue to investigate the use of existing metabolic engineering technologies and prove quick to leverage any new advances in the years to come [96, 97].

## Conclusion

In the preceding pages, we have discussed an array of site-specific bioconjugation strategies that are predicated on two simple functionalities: cysteine residues and glycans. Of course, each approach has its own intrinsic advantages and disadvantages. For example, while the modification of natural cysteine residues is both modular and straightforward, it does not offer the same degree of homogeneity and stoichiometric control as other approaches. Conversely, conjugation to *engineered* cysteine residues provides an exquisite level of stoichiometric and regiochemical control but requires genetic engineering, which limits its modularity and broad applicability. Likewise, while the modification of glycans *via* bioorthogonal click chemistry is modular, facile, and straightforward, the usefulness of this approach is necessarily limited to immunoglobulins with pendant sugar chains. Setting specifics aside, however, each of these strategies offers a route to immunoconjugates that are more homogenous and better defined than constructs created using traditional bioconjugation techniques. Furthermore, preclinical studies have shown that these site-specifically labeled immunoconjugates often boast superior *in vivo* behavior compared to their randomly constructed cousins. Somewhat curiously, while the clinical validation of site-specifically labeled immunoconjugates is of the utmost importance, work in this area seems to have stalled just short of the clinic. In our humble opinion, the move from bench to bedside is *the* most pressing imperative for the field.

In closing, it is important to note that in this installment of the review, we have only covered *two* of the *four* families of site-specific modification strategies. In Part 2 of this work, which will appear in the next issue of the journal, we will shift gears and discuss bioconjugation approaches based on peptide tags and unnatural amino acids, methods which elegantly harness chemoselective ligations and enzymatic transformations to create site-specifically modified immunoconjugates.

## Abbreviations

2-keto-Gal: 2-Acetyl-2-deoxy-galactose
ADC: Antibody-drug conjugate
ALCAM: Activated leukocyte cell adhesion molecule
CEA: Carcinoembryonic antigen
CDR: Complementarity determining region
cysDb: Cys-diabody
Db: Diabody
DBCO: Dibenzocyclooctyne
DFO: Desferrioxamine
DHZ: Dihydroazinophthalazine
DO3A: 1,4,7-Tris(carboxymethylaza)cyclododecane-10-azaacetylamide
Dol10: Dolstatin E
DOTA: 1,4,7,10-Tetraazacyclododecane-1,4,7,10-tetraacetic acid
DTPA: Diethylenetriaminepentaacetic acid
DTT: Dithiothreitol
EDTA: Ethylenediaminetetraacetic acid
GalNAz: *N*-azido-acetylgalactosamine
GalT: Galactosyltranferase
GlcNAc: *N*-acetylglucosamine
HER2: Human epidermal growth factor receptor 2
mAb: Monoclonal antibody
Mb: Minibody
MEA: Mercaptoethylamine
MHC: Major histocompatibility complex
MMAE: Monomethyl auristatin E
NCS: Isothiocyanate
NHS: *N*-hydroxysuccinimide
OI: Optical imaging
PEG: Polyethyleneglycol
PET: Positron emission tomography
PHESELECTOR: Phage ELISA for selection of reactive thiols
PSMA: Prostate-specific membrane antigen
scFv: Single-chain variable fragment
sdAb: Single-domain antibody
SialT: Sialyltransferase
SPECT: Single photon emission computed tomography
TCEP: Tris(2-carboxyethyl)phosphine
TETA: 1,4,8,11-Tetraazacyclotetradecane-1,4,8,11-tetraacetic acid
TMT-NCS: 4-(3-Isothiocyanate-4-methoxyphenyl)-6,6″-bis[*N,N*-di(carboxymethylaminomethyl]-2,2′:6′,2″-terpyridine
TNC: Tenascin-C
UDP: Uridine diphosphate
